# Accelerating 4D image reconstruction for magnetic resonance-guided radiotherapy

**DOI:** 10.1016/j.phro.2023.100484

**Published:** 2023-08-20

**Authors:** Bastien Lecoeur, Marco Barbone, Jessica Gough, Uwe Oelfke, Wayne Luk, Georgi Gaydadjiev, Andreas Wetscherek

**Affiliations:** a Joint Department of Physics at The Institute of Cancer Research and The Royal Marsden NHS Foundation Trust, 15 Cotswold Rd, London SM2 5NG, United Kingdom; b Department of Computing, Imperial College London, Exhibition Rd, South Kensington, London SW7 2BX, United Kingdom; c Department of Radiotherapy at the Royal Marsden NHS Foundation Trust, Downs Rd, London SM2 5PT, United Kingdom; d Bernoulli Institute, University of Groningen, Nijenborgh 9, Groningen 9747 AG, The Netherlands

**Keywords:** 4D-MRI, MR-guided Radiotherapy, MR-integrated Proton Therapy, Intrafraction motion, High-performance computing

## Abstract

**Background and purpose:**

Physiological motion impacts the dose delivered to tumours and vital organs in external beam radiotherapy and particularly in particle therapy. The excellent soft-tissue demarcation of 4D magnetic resonance imaging (4D-MRI) could inform on intra-fractional motion, but long image reconstruction times hinder its use in online treatment adaptation. Here we employ techniques from high-performance computing to reduce 4D-MRI reconstruction times below two minutes to facilitate their use in MR-guided radiotherapy.

**Material and methods:**

Four patients with pancreatic adenocarcinoma were scanned with a radial stack-of-stars gradient echo sequence on a 1.5T MR-Linac. Fast parallelised open-source implementations of the extra-dimensional golden-angle radial sparse parallel algorithm were developed for central processing unit (CPU) and graphics processing unit (GPU) architectures. We assessed the impact of architecture, oversampling and respiratory binning strategy on 4D-MRI reconstruction time and compared images using the structural similarity (SSIM) index against a MATLAB reference implementation. Scaling and bottlenecks for the different architectures were studied using multi-GPU systems.

**Results:**

All reconstructed 4D-MRI were identical to the reference implementation (SSIM > 0.99). Images reconstructed with overlapping respiratory bins were sharper at the cost of longer reconstruction times. The CPU  + GPU implementation was over 17 times faster than the reference implementation, reconstructing images in 60 ± 1 s and hyper-scaled using multiple GPUs.

**Conclusion:**

Respiratory-resolved 4D-MRI reconstruction times can be reduced using high-performance computing methods for online workflows in MR-guided radiotherapy with potential applications in particle therapy.

## 1 Introduction

The motion of treatment targets and vital organs poses challenges for accurately delivering external beam radiotherapy. Being able to acquire magnetic resonance imaging (MRI) with excellent soft-tissue contrast [1] at the time of treatment and resolving motion with 4D-MRI were key driving forces behind the development of MR-Linacs [2], [3], [4], [5] and hybrid MR-integrated proton therapy [6], [7].

In respiratory-resolved 4D-MRI, images are sorted based on the phase of the respiratory cycle during which they were acquired (called respiratory bin [8]), and one 3D image is reconstructed per bin. Radial acquisitions enable the extraction of a respiratory signal using self-gating [9] and reduce the blurring caused by respiratory motion [10] which led to their frequent use in 4D-MRI [10], [11], [12], [13].

To integrate 4D-MRI into clinical workflows, data are undersampled, which causes image artefacts when reconstructing directly with the Non-Uniform Fast Fourier Transform (NUFFT) [14]. Computationally-expensive iterative reconstructions based on compressed sensing [15] can mitigate the impact of undersampling by exploiting sparse data representations. Mickevicius and Paulson compared 4D-MRI reconstruction methods [16] in terms of overall image quality, reconstruction time, artefacts prevalence, and correctness of motion estimates. They found that the extra-dimensional golden-angle radial sparse parallel (XD-GRASP) algorithm [11] was least sensitive to undersampling artefacts, but they opted for the faster conjugate gradient sensitivity encoding method when implementing 4D-MRI driven MR-guided online adaptive radiotherapy [17].

In online adaptive radiotherapy, images acquired on the MR-Linac directly before the treatment are used to adapt the radiotherapy plan to the daily anatomy and target motion. For abdominal tumours affected by respiration, treatment margins can be derived from a prior 4D computed tomography, but confidence could be improved if margins were based on 4D-MRI acquired online [18], [19], [20]. In this setting, the MRI reconstruction time is critical and needs to be kept within two minutes [21], [22]. While MR-guided online adaptive proton therapy setups are still under development, early studies suggest that workflow requirements will be similar to MR-guided radiotherapy and that 4D-MRI will be necessary to account for interplay effects [23], [24].

4D-MRI could also inform clinical decisions in MR-guided interventions in the thoracic and abdominal areas such as cardiac radiofrequency catheter ablation [25] and renal and hepatic cryoablation [26].

This study aims at reducing the reconstruction time of XD-GRASP to enable the use of high-quality 4D-MRI for online-adaptive radiotherapy workflows and interventions. We propose new optimized open-source implementations of XD-GRASP leveraging acceleration for central processing units (CPUs) and graphics processing units (GPUs) and minimising both multi-threading and communication overheads by exploiting the parallel characteristics of the XD-GRASP algorithm.

## 2 Material and Methods

### 2.1 Data acquisition

Four cancer patients with pancreatic adenocarcinoma, aged between 56 and 76, two males and two females who consented to participate in the PRIMER trial (NCT02973828) [27] were imaged on a 1.5T MR-Linac (Elekta AB, Stockholm, Sweden) at different time points of their radiotherapy treatment (three patients - 36–40 Gy/ 15 fractions and one patient - 50 Gy/28 fractions). In total, 17 datasets were acquired using the vendor-provided coils (four anterior and four posterior channels). In nine of these, the patients wore an abdominal compression belt which is used for the radiotherapy treatment of pancreatic cancer at our institution. Following the current clinical protocol and similar to [17], a volumetric radial stack-of-stars gradient echo sequence with golden angle spacing [28] and balanced MRI contrast was used, which acquired 831 radial spokes in 287 s. SPAIR fat-suppression was used in combination with a partial Fourier factor of 0.7 and an oversampling factor of 1.61 along the Cartesian $k_{z}$ dimension. Partial echo sampling was used to reduce the echo time to 1.34ms. Further MRI parameters were: repetition time 3.5ms, field-ofview $500\times 500\times 200{\mathrm{mm}}^{3}$, flip angle $40^{\circ }$, bandwidth 866Hz/px, acquired voxel size $1.5\times 1.5\times 3.0{\mathrm{mm}}^{3}$. A gradient delay correction was applied [29] and coil sensitivities were estimated [30] before 4D-MRIs were reconstructed. The acquisition parameters were identical to the clinical protocol used for daily volumetric re-contouring and re-planning of MR-guided pancreatic cancer treatments at our institution [31].

### 2.2 XD-GRASP parallelisation

Before executing the XD-GRASP algorithm [11], data was prepared and arranged in a suitable format. The acquired data was first sorted into respiratory phases (bins) based on the central projection ($k_{x}=k_{y}=0$) of the k-space data. The respiratory signal was extracted by principal component analysis (PCA) of the central points and combined across the different coils. The respiratory-resolved images *d* were then reconstructed by solving Eq. 1 using conjugate gradient descent:

(1) $$d={\mathrm{argmin}}_{d}\{||F\cdot S\cdot d-m|{|}_{2}^{2}+\lambda ||T\cdot d|{|}_{1}\}$$

where consistency with the raw data *m* was enforced by applying the NUFFT operator *F* with coil sensitivities *S*. Undersampling artefacts were suppressed by regularisation with the total-variation operator [32] *T* acting along the respiratory bin dimension. The XD-GRASP algorithm reconstructed a 4D image with dimensions $n_{x},n_{y}$ and $n_{z}$ from a pre-sorted 5D signal with $n_{\mathrm{bins}}$ respiratory bins, where for each of the $n_{c}$ coil channels raw data with dimensions ${\mathrm{nk}}_{x},n_{\mathrm{lines}}={\mathrm{nk}}_{y}/n_{\mathrm{bins}}$ and ${\mathrm{nk}}_{z}$ (Eq. 2) were acquired:

(2) $$\mathrm{XD}-\mathrm{GRASP}:\mathrm{data}\{{\mathrm{nk}}_{x},n_{\mathrm{lines}},{\mathrm{nk}}_{z},n_{\mathrm{bins}},n_{c}\}\to \mathrm{images}\{n_{x},n_{y},n_{z},n_{\mathrm{bins}}\}$$

**Fig. 1:**
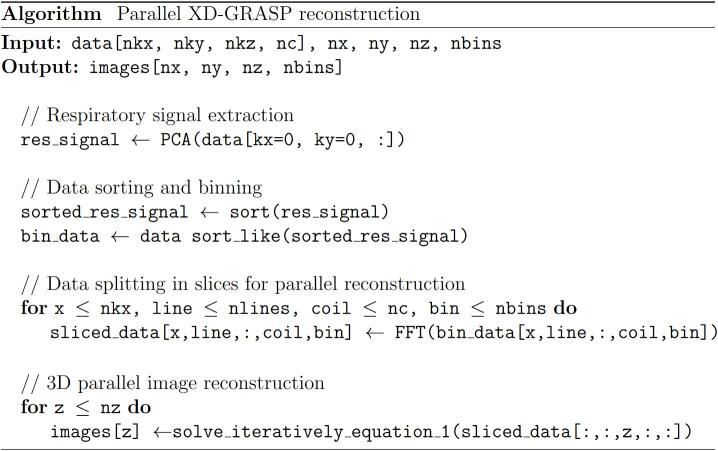
Parallel implementation of the XD-GRASP algorithm. After extraction of the respiratory signal and sorting of the data, a 1-dimensional fast Fourier transform is performed along the Cartesian dimension, to enable parallel solving of Eq. 1.

To achieve higher performance, slices were reconstructed in parallel using the algorithm from Fig. 1 by applying a Cartesian Fast Fourier Transform (FFT) along the z-axis to compute each output slice independently. This formulation is efficient because there is no communication between reconstruction instances of different slices and the computed cost function is local and benefits from early termination.

Alternative 3D formulations where the outermost loop iterates over respiratory phases are less efficient as they require sharing the NUFFT operators or communications between reconstruction instances to compute the total-variation across the different respiratory phases, which lower the achievable performance according to Amdahl’s law [33].

### 2.3 Implementation details

In Eq. 1, the NUFFT is the most computationally expensive operation with a complexity of $O({\mathrm{MJ}}^{2}+\mu^{2}N\ln(\mu^{2}N))$ [34], where *M* is the number of k-space samples acquired, *N* the pixel-size of the reconstructed image, *J* and μ the oversampling factor and the regridding kernel size used in the NUFFT implementation. The complexity for the total variation operator is lower: O(N) using the same notation as before. The NUFFT algorithm was extensively studied and optimized, hence this study leverages the state-of-the-art *FINUFFT* [35] and *cuFINUFFT* [36] libraries. XD-GRASP is an *iterative* conjugate-gradient algorithm, requiring multiple NUFFT operations both from non-uniform k-space to a uniform image (type one) and from image space back to k-space (type two). We handled the parallelisation manually using OpenMP [37] multi-threading capabilities to decrease the threading overhead and reuse the same thread for multiple slices. In the GPU case, this allowed us to saturate the GPU memory and reuse the data already transferred to the GPU and hide data transfer latency by overlapping computations and transfers.

When computing the NUFFT, interpolation-based implementations require evaluating a Cartesian FFT on a fine uniform grid oversampled by a factor of *R*. For a fixed arbitrary calculation precision, multiple oversampling factors can be used [38] and we tested reconstructions with R=2 and R=1.25. At the time of writing, cuFINUFFT did not provide pre-computed weights for R=1.25, we therefore limited the GPU experiments to R=2. Following a previous study [39], we selected a NUFFT approximation error below $10^{-3}$, leading to kernel widths of four for R=2 and five for R=1.25 [35]. The planning interface of FINUFFT and cuFINNUFT was used, such that NUFFT operators were only computed once per type (type one and type two) and per thread and could be reused across slices.

### 2.4 Reconstruction experiments

Strategies used to bin the data could affect the reconstructed images depending on the mapping from data to respiratory phases [10] by reducing the undersampling or by considering a more robust respiratory surrogate. These changes can lead to a difference in reconstruction times by altering the convergence of the algorithm, especially if the amount of data per respiratory bin is modified. Thus, we extracted respiratory signals using two self-gating methods: the baseline strategy described in the XD-GRASP study [11] and a self-gating strategy applying an angle-dependent correction of the respiratory signal (ADERS) [8]. In the ADERS strategy, the magnitude signal of the k-space centre is sorted by acquisition angle and a background subtraction based on the moving average across adjacent angles is performed. After this correction, the quality of the respiratory signal is estimated for each receive coil [40] and half of the coils with the lowest data quality are rejected before applying PCA across the remaining coils. For a given respiratory signal, the acquired data is divided either into respiratory bins [11] or assigned to overlapping respiratory bins (ORB), in which a single spoke contributes to two adjacent respiratory bins, except for the extremum bins corresponding to maximum inhalation and exhalation. We combined the two binning techniques with the two self-gating methods, resulting in four data preparation strategies (baseline, ADERS, ORB, combined).

To evaluate the reconstruction speed, volumetric images with 336×336×64 voxels across 8 respiratory phases were reconstructed using 8 iterations of XD-GRASP with regularisation parameter λ=0.02 for all acquisitions and data preparation strategies. Each experiment was repeated five times and results were averaged across the runs. Images obtained with the fast CPU/ CPU-GPU implementations were compared with a parallelised MATLAB implementation based on Feng et al. [11] using the Structural Similarity Index Measure (SSIM) [41]. The reference MATLAB implementation relied on the same inter-slice parallelisation but did not use the FINUFFT library. To compare the effect of integrating the FINUFFT library directly into the MATLAB code, a fifth parallel version was implemented using the available FINUFFT CPU bindings in MATLAB with an oversampling factor of R=1.25.

### 2.5 Open-source framework

The proposed fast reconstruction framework (source code available on. https://github.com/instituteofcancerresearch) can perform all the necessary steps to reconstruct images from raw data, including respiratory signal extraction and data sorting. To facilitate the adoption of the framework, a MATLAB interface is provided, enabling users to change reconstruction parameters and to visualise and export results.

### 2.6 Scaling experiments

For the reconstruction experiments above, the timings were evaluated on a high-end consumer system incorporating an Intel i9-11900K 8-core CPU, DDR4-2933 MHz random access memory (RAM) and an NVIDIA RTX A5000 GPU using FINUFFT v2.0.4 (commit 0e013e6) and cuFINUFFT v1.2 (commit c17b3a9). The C++ and CUDA codes were compiled with GCC 11.1.0 and NVCC 11.4, respectively, and leveraged the AXV512 long vector instructions available on the CPU.

To test the bottlenecks and the scaling of our accelerated implementations, a single dataset from this study, previously used in [42] was reconstructed with the baseline strategy using two other systems - an AMD Ryzen 3700X CPU with DDR4-3600 MHz RAM for the R=1.25 CPU version and a multi-GPU system containing three NVIDIA 1080Ti GPUs for the GPU version. To benefit from the multiple GPUs on the multi-GPU system, the slices were distributed equally across the available GPUs such that each GPU reconstructed adjacent slices. The observed scaling was compared to the theoretical linear scaling. To evaluate the scaling dependency on the number of respiratory phases, the same dataset was reconstructed using the Intel system while varying the number of bins.

### 2.7 Amdhal’s law

The theoretically achievable times were estimated using Amdahl’s law [33] for our CPU and GPU models using Eqs. (3.a), (3.b), assuming that the number of CPU cores was higher or equal to the number of slices to reconstruct.

(3.a) $$T_{\mathrm{XD}-\mathrm{GRASP},\mathrm{CPU}}=T_{\mathrm{Preparation}}+T_{\mathrm{Threading}}+T_{\mathrm{Plan}}+\max(T_{\mathrm{Slice}})$$

(3.b) $$T_{\mathrm{XD}-\mathrm{GRASP},\mathrm{GPU}}=T_{\mathrm{Preparation}}+T_{\mathrm{Transfers}}+\max(T_{\mathrm{slice}})$$

In the CPU case, the reconstruction time is governed by the slice with the longest reconstruction time ($\max(T_{\mathrm{slice}})$). Using the same data as for our scaling experiment, we measured the reconstruction time for each slice. In Eq. 3.b, the NUFFT pre-calculations are masked by the time to transfer the data from CPU to GPU ($T_{\mathrm{Transfers}}$). In the GPU case, we can further assume that $T_{\mathrm{Slice}}\approx 0$ for all slices reconstructed simultaneously on the GPU assuming infinite parallelism.

## 3 Results

### 3.1 Reconstructed images

**Fig. 2:**
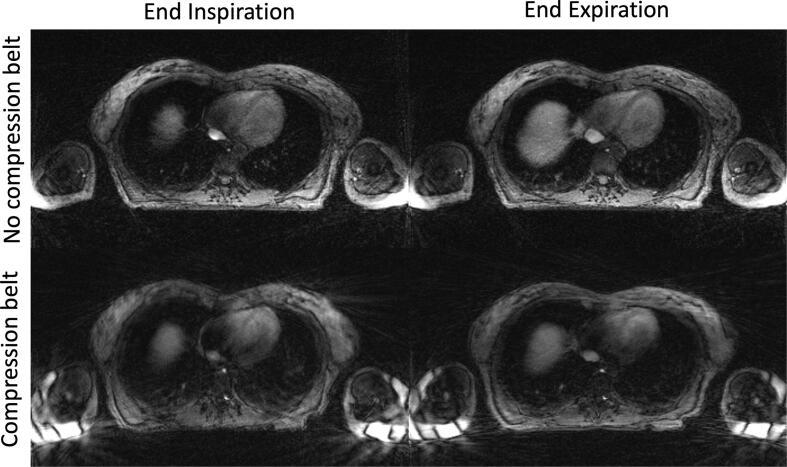
Slice reconstructed for the end inspiration and end respiration phases. The differences in respiration can be assessed using the size of the liver dome in both images and show a larger motion amplitude when no compression belt was applied. Streaking artefacts originating from the arms are visible at the edge of the field-of-view, but do not affect the excellent visualisation of the internal anatomy.

**Fig. 3:**
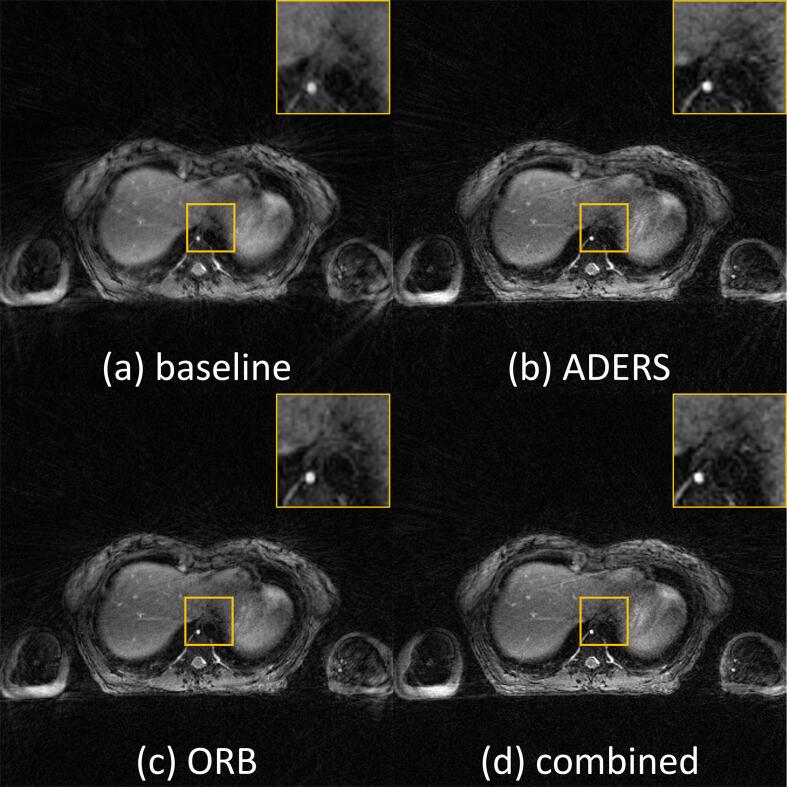
End-inspiratory phase of 4D-MRI acquired with abdominal compression and reconstructed from the same acquisition using the different data preparation strategies. Details from fine non-moving structures appear sharper in (b), (c) and (d) compared with the baseline (a).

4D-MRIs reconstructed using XD-GRASP showed remaining streaking artefacts, primarily originating from the arms. They did not affect the excellent visualisation of the internal anatomy with and without the compression belt (Fig. 2, Fig. 3). Images reconstructed with the proposed fast implementations were identical to the reference MATLAB implementation, with each reconstruction achieving an SSIM of 0.99. Images acquired with a compression belt appeared more blurry when using the baseline data preparation strategy compared to the other methods. In particular, non-moving structures appeared sharper when more data per bin was used (ORB and combined) as seen in Fig. 3.

### 3.2 Measured speed-ups

**Fig. 4:**
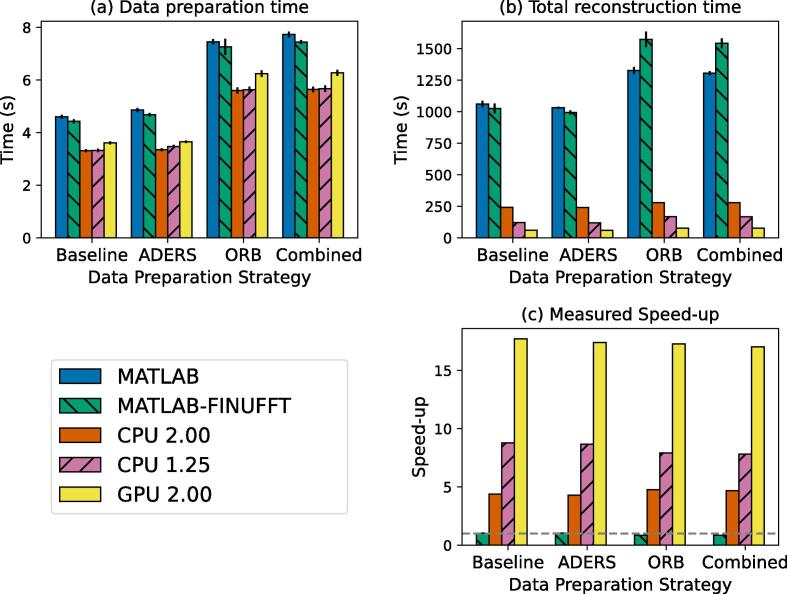
Timing and speed-up results for the different data preparation strategies using our different implementations. The ORB and combined data preparation strategies increased both the data preparation (a) and the overall reconstruction times (b). In all cases, the GPU implementation was more than 17 times faster than the MATLAB reference implementation (c), reconstructing images in 60s (baseline, ADERS) and 77s (ORB, combined). The MATLAB implementation using the FINUFFT bindings was slower than the original version for ORB and combined with speed-up below 1 (dashed line).

Speed-ups using the C++ implementations can be observed during the data preparation, (Fig. 4a) when compared with the MATLAB implementations. This part of the code had no GPU acceleration. The ADERS strategy showed no timing difference from the baseline. The ORB and combined strategies required longer preparation and overall reconstruction times (Fig. 4b). Non-overlapping bins (baseline, ADERS) were reconstructed in 1060 ± 26 s with MATLAB, 1020 ± 40 s for the MATLAB-FINUFFT, 241 ± 3.3 s with CPU and R=2.0, 120 ± 3 s with CPU and R=1.25, and in 59 ± 1 s with the heterogeneous CPU  + GPU implementation. Reducing the oversampling factor from R=2.0 to R=1.25 halved the reconstruction time for our CPU-only implementation. The GPU version achieved the best speed-up (Fig. 4c), being more than 17x faster than the baseline version.

### 3.3 Scaling

**Fig. 5:**
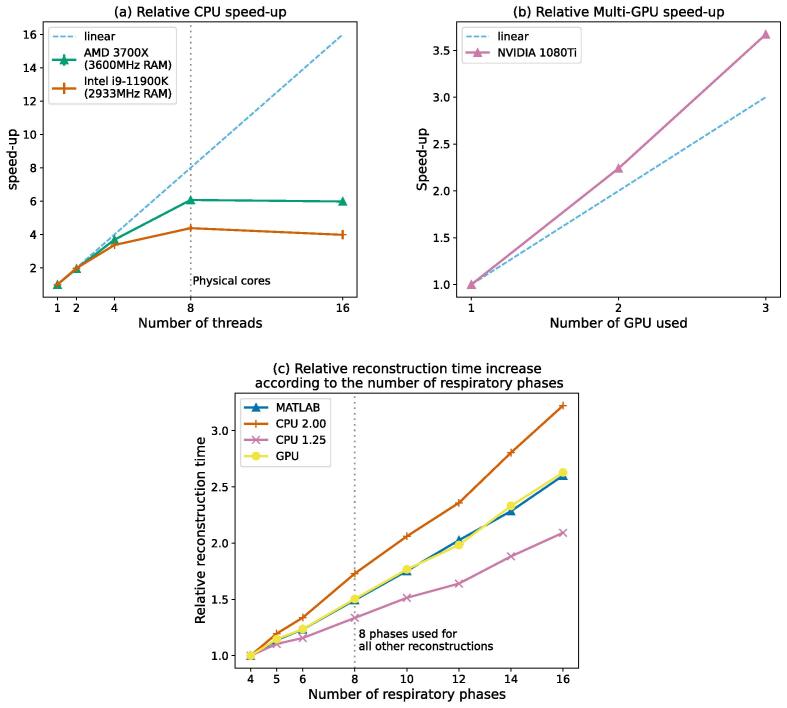
The straight line represents the theoretical linear speed-up for the number of threads (a) and the number of GPUs used (b). The CPU scaling experiment showed less than optimal scaling on both testing systems though the system with higher RAM speed achieved higher scaling. In the GPU case, a hyper-scaling phenomenon was observed where the observed speed-up was higher than the linear speed-up. Reconstruction time scaled linearly with the number of respiratory phases (c).

The CPU implementation showed sub-linear scaling on both tested systems (Fig. 5a) with similar single-core performance. The reconstruction benefited from a higher thread count up to the number of physical cores of the tested machines. The system with the higher RAM bandwidth showed better scaling at higher thread counts. The measured speed-up on the system with multiple GPUs, represented in Fig. 5b, was higher than the theoretical linear speed-up with the reconstruction being 3.6 times faster when using three GPUs. This could be attributed to a better resource utilisation across the CPU and GPU. The reconstruction time increased linearly with the number of respiratory phases for all implementations.

### 3.4 Amdhal’s law

Following the assumptions of Eqs. 3.a and 3.b, we measured that the minimal achievable reconstruction time for our datasets would be $9.42s+T_{\mathrm{Preparation}}$ for the CPU only reconstruction and $1.95s+T_{\mathrm{Preparation}}$ for the GPU case. In the GPU case, the data preparation time on the CPU would be larger than the slice reconstruction time on the GPU.

## 4 Discussion

Short reconstruction times are required to integrate advanced reconstruction methods such as 4D-MRI into online adaptive radiotherapy workflows. By applying parallelisation and high performance computing, our open-source implementations shorten the reconstruction time of 4D-MRI while maintaining the same image quality. Using a GPU, we achieved reconstruction times of one minute.

Time constraints for online adaptive radiotherapy are loosely defined in literature. Feng et al. suggested that online treatment adaptation requires a reconstruction time below two minutes for typical matrix sizes of 256×256×48 voxles and 10 respiratory phases [22], which is equivalent to a minimal throughput of 262,144pixels/second. Our fast CPU and GPU implementations exceeded this minimum throughput and are therefore promising candidates for MR-guided online-adaptive radiotherapy. We compared our implementations to other state-of-the-art XD-GRASP implementations based on published information (Supplementary Document 1). Most of the previous studies did not meet the throughput requirement except for the CPU + GPU implementation from Barbone et al. [42], which was faster than all our implementations. However, they achieved a lower relative speed-up of 11x and SSIM of 0.97 compared to the same reference MATLAB implementation, using a high-end enterprise-grade system containing two AMD EPYC^TM^ 7551 and an NVIDIA TESLA V100 GPU.

Instead of re-implementing the XD-GRASP algorithm in C++/ CUDA, less involved techniques could have been considered to improve performance. We explored calling FINUFFT directly from MATLAB, but it achieved no performance gain, leading to longer reconstructions when overlapping bins were used. Another possible optimisation route would be the use of automated conversion tools, such as MATLAB Coder^TM^ to generate C++ code. However, this could have led to the duplication of interfaces, as the MATLAB reference implementation used an external library [43] for performing the NUFFT.

In all cases, the application gained in performance using multi-threading, achieving sub-linear scaling depending on the RAM speed. This is compatible with benchmarks performed by the FINUFFT library developers [35] who suggested that for a precision of $10^{-3}$, the NUFFT execution time was dominated by the RAM access times to the kernel data, instead of the theoretical operations speed of the CPU. As enterprise-grade systems with high-core counts become available in clinical settings [17], the reconstruction time for inter-slice parallelised algorithms should decrease until one slice is reconstructed per core, with speed-ups getting closer to the ones predicted by Amdahl’s law.

The number of slices to reconstruct is determined by the size of the field-of-view along the superior-inferior axis and the slice thickness. For our data, the field-of-view was 200 mm, in line with the maximum treatment field of 220 mm of the MR-Linac. Future applications may however require larger field-of-views to fully cover large organs-at-risk, such as the lungs. In this case, the best performance is obtained if the number of slices is a multiple of the number of CPU cores to balance the workload equally among cores.

We noticed a trade-off between reconstruction time and image quality when using the different data preparation strategies. While more temporal blurring could be expected when doubling the data in each respiratory bin, we found that the images reconstructed using the ORB and combined methods appeared sharper. We hypothesise that the reduced amount of undersampling artefacts combined with a lower total variation score between adjacent overlapping respiratory bins could mitigate the smoothing effects of the regularisation.

In this study, we reconstructed data acquired with a balanced contrast and fat-suppression. Clinically, however, we found that the optimal contrast is dependent on the patient. Multiple contrasts are acquired in a first session (balanced and spoiled sequences with and without fat-suppression) and the contrast offering the best tumour and organs-at-risk visibility is used throughout the treatment sessions. The use of different contrasts could affect the reconstruction times of our fast implementations.

The primary applications for respiratory-correlated 4D-MRI in MR-guided radiotherapy [10] are measuring the extent of respiratory motion for treatment planning, providing data as input for time-resolved 4D-MRI and calculating the delivered dose [44], [45]. These concepts apply to image-guided proton therapy as well, where interplay effects [7] and the Bragg peak depth could require an even lower image reconstruction latency [46].

We explored the benefits of inter-slice parallelism for the XD-GRASP algorithm. The presented approach could be applied to other MRI reconstruction algorithms, where at least one sampling dimension is Cartesian, such as the stack-of-stars [28] or the stack-of-spirals [47] patterns and could be extended to time-resolved 4D-MRI [10] based on XD-GRASP such as MRSIGMA [22] or SPIDERM [48]. In particular regarding time-resolved 4D-MRI, the use of randomized projection-encoding [49] could be beneficial, but wouldn’t allow for straight-forward inter-slice parallelism. Compared with AI-based reconstruction schemes [50], [51], our proposed implementations provided an excellent agreement with the reference implementation.

Using our open-source implementation, 4D-MRI reconstruction takes less than two minutes, even without GPU accelerator, enabling their use for online adaptive radiotherapy. Our results make 4D-MRI an attractive solution for monitoring intra-fraction motion in MR-guided radiotherapy, where the superior soft-tissue contrast compared to 4D computed tomography could reduce the uncertainty in tumour position and enable online adaptive gated treatments and real-time tracking [52] without the need for fiducial markers. This could benefit the treatment of lung, liver, pancreas and oligometastatic disease [53].

## CRediT authorship contribution statement

**Bastien Lecoeur:** Software, Investigation, Validation, Writing - original draft. **Marco Barbone:** Methodology, Software, Investigation, Writing - original draft. **Jessica Gough:** Data curation, Writing - review & editing. **Uwe Oelfke:** Supervision, Writing - review & editing. **Wayne Luk:** Conceptualization, Supervision, Writing - review & editing. **Georgi Gaydadjiev:** Conceptualization, Supervision, Writing - review & editing. **Andreas Wetscherek:** Conceptualization, Supervision, Writing - review & editing.

## Declaration of Competing Interest

The authors declare the following financial interests/personal relationships which may be considered as potential competing interests: The Institute of Cancer Research and the Royal Marsden NHS Foundation Trust are part of the Elekta MR-Linac Research Consortium. We thank Philips for partnering with us on this research and providing MR source code, research licences, and support.

## References

[1] Schmidt M.A., Payne G.S. (2015). Radiotherapy planning using MRI. Phys Med Biol.

[2] Lagendijk J.J., Raaymakers B.W., Raaijmakers A.J., Overweg J., Brown K.J., Kerkhof E.M. (2008). MRI/linac integration. Radiother Oncol.

[3] Fallone B.G., Murray B., Rathee S., Stanescu T., Steciw S., Vidakovic S. (2009). First MR images obtained during megavoltage photon irradiation from a prototype integrated linac-MR system. Med Phys.

[4] Keall P.J., Barton M., Crozier S. (2014). The Australian Magnetic Resonance Imaging-Linac Program. Semin Radiat Oncol.

[5] Mutic S., Low D., Chmielewski T., Fought G., Hernandez M., Kawrakow I. (2016). TU-H-BRA-08: The Design and Characteristics of a Novel Compact Linac-Based MRI Guided Radiation Therapy (MR-IGRT) System (abstr). Med Phys.

[6] Hoffmann A., Oborn B., Moteabbed M., Yan S., Bortfeld T., Knopf A. (2020). MR-guided proton therapy: a review and a preview. Radiat Oncol.

[7] Oborn B.M., Dowdell S., Metcalfe P.E., Crozier S., Mohan R., Keall P.J. (2017). Future of medical physics: Real-time MRI-guided proton therapy. Med Phys.

[8] Rank C.M., Heußer T., Buzan M.T., Wetscherek A., Freitag M.T., Dinkel J. (2017). 4D respiratory motion-compensated image reconstruction of free-breathing radial MR data with very high undersampling. Magn Reson Med.

[9] Paul J., Divkovic E., Wundrak S., Bernhardt P., Rottbauer W., Neumann H. (2015). High-resolution respiratory self-gated golden angle cardiac MRI: Comparison of self-gating methods in combination with k-t SPARSE SENSE. Magn Reson Med.

[10] Stemkens B., Paulson E.S., Tijssen R.H. (2018). Nuts and bolts of 4D-MRI for radiotherapy. Phys Med Biol.

[11] Feng L., Axel L., Chandarana H., Block K.T., Sodickson D.K., Otazo R. (2016). XD-GRASP: Golden-angle radial MRI with reconstruction of extra motion-state dimensions using compressed sensing. Magn Reson Med.

[12] Lustig M., Pauly J.M. (2010). SPIRiT: Iterative self-consistent parallel imaging reconstruction from arbitrary k-space. Magn Reson Med.

[13] Wright K.L., Hamilton J.I., Griswold M.A., Gulani V., Seiberlich N. (2014). Non-Cartesian parallel imaging reconstruction. J Magn Reson Imaging.

[14] Dutt A., Rokhlin V. (1993). Fast Fourier Transforms for Nonequispaced Data. SIAM J Sci Comput.

[15] Lustig M., Donoho D., Pauly J.M. (2007). Sparse MRI: The application of compressed sensing for rapid MR imaging. Magn Reson Med.

[16] Mickevicius N.J., Paulson E.S. (2017). Investigation of undersampling and reconstruction algorithm dependence on respiratory correlated 4D-MRI for online MR-guided radiation therapy. Phys Med Biol.

[17] Paulson E., Ahunbay E., Chen X., Mickevicius N., Chen G.P., Schultz C. (2020). 4D-MRI Driven MR-guided Online Adaptive Radiotherapy for Abdominal Stereotactic Body Radiation Therapy on a High Field MR-Linac: Implementation and Initial Clinical Experience. Clin Trans Radiat Oncol.

[18] Uh J., Kadbi M., Hua C.H. (2019). Effects of age-related breathing characteristics on the performance of four-dimensional magnetic resonance imaging reconstructed by prospective gating for radiation therapy planning. Phys Imaging Radiat Oncol.

[19] Perkins T., Lee D., Simpson J., Greer P., Goodwin J. (2021). Experimental evaluation of four-dimensional Magnetic Resonance Imaging for radiotherapy planning of lung cancer. Phys Imaging Radiat Oncol.

[20] Habatsch M., Schneider M., Requardt M., Doussin S. (2022). Movement assessment of breast and organ-at-risks using free-breathing, self-gating 4D magnetic resonance imaging workflow for breast cancer radiation therapy. Phys Imaging Radiat Oncol.

[21] Hunt A., Hansen V.N., Oelfke U., Nill S., Hafeez S. (2018). Adaptive Radiotherapy Enabled by MRI Guidance. Clin Oncol.

[22] Feng L., Tyagi N., Otazo R. (2020). MRSIGMA: Magnetic Resonance SIGnature MAtching for real-time volumetric imaging. Magn Reson Med.

[23] Dolde K., Zhang Y., Chaudhri N., Dávid C., Kachelrieß M., Lomax A.J. (2019). 4DMRI-based investigation on the interplay effect for pencil beam scanning proton therapy of pancreatic cancer patients. Radiat Oncol.

[24] Dowdell S., Grassberger C., Sharp G.C., Paganetti H. (2013). Interplay effects in proton scanning for lung: a 4D Monte Carlo study assessing the impact of tumor and beam delivery parameters. Phys Med Biol.

[25] Zan G., Calò L., Borrelli A., Guglielmo M., de Ruvo E., Rier S. (2023). Cardiac magnetic resonance-guided cardiac ablation: a case series of an early experience. Eur Heart J Supplements.

[26] Wang L., Liu C., Liu J., Li P., Xiang J., Liu M. (2019). MRI-Guided Cryoablation of Hepatic Dome Hepatocellular Carcinomas Using 1-T Open High-Field-Strength Scanner. AM J Roentgenolog.

[27] PRIMER: Development of Daily Online Magnetic Resonance Imaging for Magnetic Resonance Image Guided Radiotherapy. https://clinicaltrials.gov/ct2/show/NCT02973828.

[28] Block K.T., Chandarana H., Milla S., Bruno M., Mulholland T., Fatterpekar G. (2014). Towards Routine Clinical Use of Radial Stack-of-Stars 3D Gradient-Echo Sequences for Reducing Motion Sensitivity. J Korean Soc Magn Reson Med.

[29] Ianni J.D., Grissom W.A. (2016). Trajectory Auto-Corrected image reconstruction. Magn Reson Med.

[30] Walsh D.O., Gmitro A.F., Marcellin M.W. (2000). Adaptive reconstruction of phased array MR imagery. Magn Reson Med.

[31] Gough J., Wetscherek A., Lecoeur B., Alexander S., Westley R., Ng-Cheng-Hin B. (2023). PD-0658 Use of 4DMRI acquired on an MR-Linac to quantify intra-fraction motion in pancreatic cancer (abstr). Radiother Oncol.

[32] Rudin L.I., Osher S., Fatemi E. (1992). Nonlinear total variation based noise removal algorithms. Phys D.

[33] Rodgers D.P. (1985).

[34] Song J, Liu Y, Gewalt SL, Cofer G, Johnson GA, Liu* QH. Least-Square NUFFT Methods Applied to 2-D and 3-D Radially Encoded MR Image Reconstruction. IEEE Trans Bio-med Eng 2009;56:1134–42. doi: 10.1109/TBME.2009.2012721. 10.1109/TBME.2009.2012721 PMC2734456 19174334

[35] Barnett AH, Magland J, af Klinteberg L. A Parallel Nonuniform Fast Fourier Transform Library Based on an “Exponential of Semicircle” Kernel. SIAM J Sci Comput 2019;41:C479–C504. doi: 10.1137/18M120885X.

[36] Shih Yh, Wright G, Andén J, Blaschke J, Barnett AH. cuFINUFFT: a load-balanced GPU library for general-purpose nonuniform FFTs. IEEE Int Parallel Distrib P 2021;1:688–97. doi: 10.1109/IPDPSW52791.2021.00105.

[37] Dagum L., Menon R. (1998). OpenMP: an industry standard API for shared-memory programming. IEEE Comput Sci Eng.

[38] Barnett A.H. (2021). Aliasing error of the $\exp(\beta_{1}-z^{2})$ kernel in the nonuniform fast Fourier transform. Appl Comput Harmon Anal.

[39] Beatty P.J., Nishimura D.G., Pauly J.M. (2005). Rapid gridding reconstruction with a minimal oversampling ratio. IEEE Trans Med Imaging.

[40] Grimm R, Bauer S, Kiefer B, Hornegger J, Block T. Optimal Channel Selection for Respiratory Self-Gating Signals (abstr). in: Proc Intl Soc Mag Reson Med 21. 2013. p. 3749.

[41] Wang Z., Bovik A.C., Sheikh H.R., Simoncelli E.P. (2004). Image quality assessment: From error visibility to structural similarity. IEEE Trans Image Process.

[42] Barbone M., Wetscherek A., Yung T., Oelfke U., Luk W., Gaydadjiev G. (2021). Efficient Online 4D Magnetic Resonance Imaging. Int Sym Comp Archit.

[43] Fessler J., Sutton B. (2003). Nonuniform fast fourier transforms using min-max interpolation. IEEE Trans Signal Proces.

[44] Stemkens B., Tijssen R.H.N., de Senneville B.D., Lagendijk J.J.W., van den Berg C.A.T. (2016). Image-driven, model-based 3D abdominal motion estimation for MR-guided radiotherapy. Phys Med Biol.

[45] Menten M.J., Mohajer J.K., Nilawar R., Bertholet J., Dunlop A., Pathmanathan A.U. (2020). Automatic reconstruction of the delivered dose of the day using MR-linac treatment log files and online MR imaging. Radiother and Oncol.

[46] Goodburn R.J., Philippens M.E.P., Lefebvre T.L., Khalifa A., Bruijnen T., Freedman J.N. (2022). The future of MRI in radiation therapy: Challenges and opportunities for the MR community. Magn Reson Med.

[47] Lee J.H., Hargreaves B.A., Hu B.S., Nishimura D.G. (2003). Fast 3D imaging using variable-density spiral trajectories with applications to limb perfusion. Magn Reson Med.

[48] Han P., Chen J., Xiao J., Han F., Hu Z., Yang W. (2022). Single projection driven real-time multi-contrast (SPIDERM) MR imaging using pre-learned spatial subspace and linear transformation. Phys Med Biol.

[49] Subashi E., Feng L., Liu Y., Robertson S., Segars P., Driehuys B. (2023). View-sharing for 4D magnetic resonance imaging with randomized projection-encoding enables improvements of respiratory motion imaging for treatment planning in abdominothoracic radiotherapy. Phys Imaging Radiat Oncol.

[50] Freedman J.N., Gurney-Champion O.J., Nill S., Shiarli A.M., Bainbridge H.E., Mandeville H.C. (2021). Rapid 4D-MRI reconstruction using a deep radial convolutional neural network: Dracula. Radiother Oncol.

[51] Terpstra M.L., Maspero M., Verhoeff J.J.C., van den Berg C.A.T. (2023;:2023. 1.). Accelerated respiratory-resolved 4D-MRI with separable spatio-temporal neural networks. Med Phys.

[52] Bert C., Saito N., Schmidt A., Chaudhri N., Schardt D., Rietzel E. (2007). Target motion tracking with a scanned particle beam. Med Phys.

[53] Pham T.T., Whelan B., Oborn B.M., Delaney G.P., Vinod S., Brighi C. (2022). Magnetic resonance imaging (MRI) guided proton therapy: A review of the clinical challenges, potential benefits and pathway to implementation. Radiother Oncol.

