# Supplementary material for: Accelerating 4D image reconstruction for magnetic resonance-guided radiotherapy
Source: Phys Imaging Radiat Oncol. 2023 Aug 20;27:100484. doi: 10.1016/j.phro.2023.100484 (PMC10474606; doi:10.1016/j.phro.2023.100484)
Supplement: Supplementary data 1 [file mmc1.docx]

Supplementary Materials

Performance comparison with other implementations


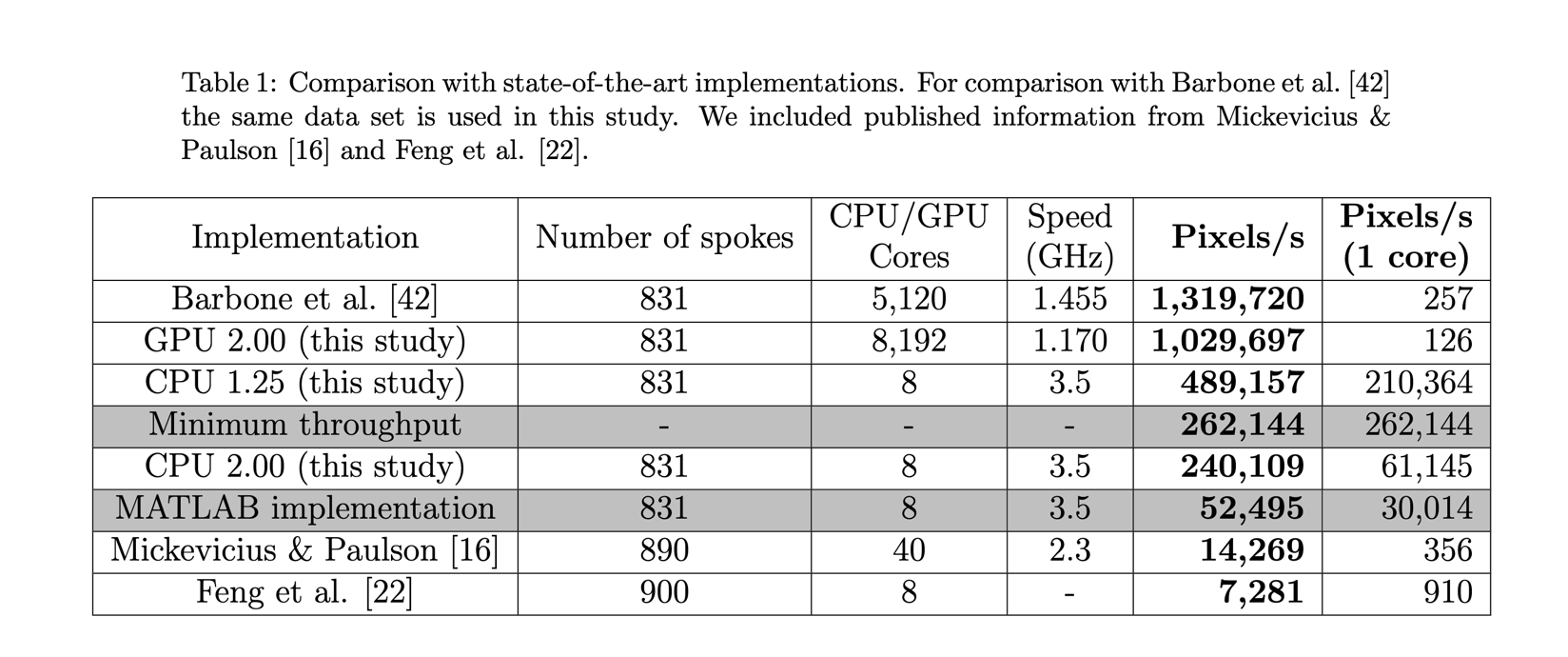


Supplementary Table 1: Comparison with state-of-the-art implementations. For comparison with Barbone et al. [42] the same dataset is used in this study. We included published information from Mickevicius & Paulson [16] and Feng et al. [22]

# 48-core scaling experiment

The CPU scaling experiment performed on commercial-grade systems (see section X.Y in the main manuscript) was repeated on a high-end system with 48 cores using 2 Intel Xeon Platinum 8260(Cascade Lake) processors and DDR4-2933MHz RAM. Supplementary Figure 1 shows the results. The speed-up was consistent with the results obtained with the two other systems, showing sub-linear scaling up to the number of physical cores of the machine.

A potential explanation for the sub-linear speed-up is that the number of reconstructed slices was 64, which leads to a load imbalance when using 24 and 48 cores. In this case, some threads will reconstruct 2 slices consecutively while the other will only reconstruct a single slice.

Supplementary Figure 1: Reconstruction speed-up when using a high number of physical cores. Scaling is sub-linear up to the number of physical cores of the machine.

# Respiratory signals extraction details

We present details on the more robust respiratory signal extraction technique, which uses an angle-dependent correction (ADERS).

In this method, the spokes are first sorted according to the angle at which they were acquired. Then, a background magnitude signal is computed by binning the spokes by angle (with each bin of size 1^o^) and averaging the magnitude of all spokes acquired within the bin. The spokes are then corrected by removing the background signal as the moving average across the different acquisition angles. This removes the angle dependency of the respiratory signal. The data from the best coils are then selected to produce the respiratory surrogate: the frequency content of each coil is estimated in terms of respiratory and cardiac frequency ranges following [40]. The best half of the coils is selected to compute the surrogate signal. The signal is then smoothed per coil along its temporal dimension using a moving average filter of width 3. To combine the signals from the different coils, a Principal Component Analysis (PCA) is finally applied, and a single respiratory signal is derived.

Supplementary Figure 2: Respiratory signals extracted from two scans realised on the same patient without and with the use of the compression belt. For each of the two dataset the respiratory signal is extracted using the different methods. The two signals appear similar when the compression belt is not used.
